# The pan-immune-inflammation value and systemic immune-inflammation index in advanced melanoma patients under immunotherapy

**DOI:** 10.1007/s00432-021-03878-y

**Published:** 2022-01-10

**Authors:** L. Susok, S. Said, D. Reinert, R. Mansour, C. H. Scheel, J. C. Becker, T. Gambichler

**Affiliations:** 1 Skin Cancer Center, Department of Dermatology, Ruhr-University Bochum, Bochum, Germany; 2 Department of Radiology, Ruhr-University Bochum, Bochum, Germany; 3 Translational Skin Cancer Research, DKTK Partner Site Essen/Düsseldorf, West German Cancer Center, Dermatology, University Duisburg-Essen, Essen, Germany; 4 German Cancer Research Center (DKFZ), Heidelberg, Germany

**Keywords:** Cutaneous melanoma, Immune checkpoint inhibitors, Ipilimumab, Pembrolizumab, Nivolumab, Pan-immune-inflammation value, Systemic immune-inflammation index

## Abstract

**Purpose:**

To evaluate the pan-immune-inflammation value (PIV) and systemic immune-inflammation index (SII) in patients with cutaneous melanoma (CM) under immune checkpoint inhibitor (ICI) therapy.

**Methods:**

PIV and SII were calculated before the start of ICI therapy and at time of progression/death in patients with metastatic CM (stage III/IV). Sex–age-matched CM patients in stage I/II and healthy subjects (HC) served as controls.

**Results:**

The median PIV of stage III/IV patients was significantly (*P* = 0.0011) higher than in stage I/II patients and HC. SII was significantly (*P* = 0.00044) lower in HC than in CM patients. At baseline, PIV and SII did significantly correlate with lactate dehydrogenase (*P* = 0.045/0.017). However, ROC curve statistics revealed that SII and PIV were not significantly associated with clinical parameters, including best response to ICI treatment (*P* = 0.87/0.64), progression-free survival (*P* = 0.73/0.91), and melanoma-specific survival (*P* = 0.13/0.17). Moreover, there were no significant changes of PIV and SII from baseline to progression/death (*P* = 0.38/0.52).

**Conclusions:**

Even though both immune-inflammation biomarkers showed some power to differentiate between CM stages and HC, respectively, PIV and SII seem not to be significant predictors for clinical outcome measures of CM patients under ICI therapy.

## Introduction

In Caucasians, incidences of cutaneous melanoma (CM) are increasing worldwide, with estimated continuous case increases for the next decades. The highest incidence is found in Queensland, Australia (about 70 cases/100.000/year). In the USA, an increasing incidence from 14 to 22/100.000 person-years has been observed across all primary tumor thicknesses. Similarly, the incidence of invasive MM increases in Europe mostly attributed to the increasing incidence of thin melanomas (Whiteman et al. 2016). Importantly, more than 55.000 deaths per year can be attributed to CM worldwide. Immune checkpoint inhibitors (ICI), including the programmed death protein 1 (PD-1, pembrolizumab, nivolumab) and cytotoxic T lymphocyte associated protein 4 (CTLA-4, ipilimumab), recently turned out to be effective in melanoma treatment. Unfortunately, approximately 50% of patients do not respond to ICI and it is still difficult to predict who will respond to these agents. Thus, there is high need for potent biomarkers predicting the treatment outcome to ICI, in particular considering ICI-mediated adverse events and high cost (Whiteman et al. 2016; Seité et al. 2017; Marconcini et al. 2018; Schadendorf et al. 2018).

There is growing evidence that systemic inflammatory responses represent significant determinants of tumor progression and survival in many malignancies. Hence, several immune-based prognostic scores, such as neutrophil count, lymphocyte count, neutrophil/lymphocyte ratio (NLR), platelet/lymphocyte ratio (PLR), and monocyte/lymphocyte ratio (MLR), have been employed to predict the prognosis in several cancers, including CM (Zaragoza et al. 2016; Wade et al. 2018; Robinson et al. 2020; Bai et al. 2021; Hernando-Calvo et al. 2021; Ludwig et al. 2021). However, there are novel, more complex complete blood count-based immune-inflammation biomarkers, such as the systemic immune-inflammation index (SII) and the pan-immune-inflammation value (PIV), which have not yet been investigated thoroughly in patients with CM and other skin cancers (Whiteman et al. 2016; Yang et al. 2018; Fucà et al. 2020; Hernando-Calvo et al. 2021). In the present study, we aimed to investigate PIV and SII in control subjects and patients with metastatic CM who underwent ICI treatment.

## Methods

### Patients

This study was performed at the Skin Cancer Center of the Ruhr-University Bochum (Bochum, Germany). It was conducted according to the declaration of Helsinki and followed a protocol approved by our institutional ethics review board (#16-5985). We studied patients with inoperable stage III or IV CM who had the indication for ICI treatment. Therapy and staging procedures were performed in accordance with national guidelines for the management of CM and interdisciplinary tumor board decisions (Schadendorf et al. 2018). ICI, including mono-nivolumab, mono-pembrolizumab, ipilimumab, ipilimumab plus nivolumab, was administered in label (Marconcini et al. 2018). Complete work-up was regularly performed including lymph node ultrasound, thoracic and/or abdominal computed tomography (CT) or positron emission tomography in combination with computer tomography (PET-CT), and cranial magnetic resonance tomography (Schadendorf et al. 2018). The criteria for treatment response were used in accordance with RECIST 1.1 (Eisenhauer et al. 2009). To rule out pseudo-progress, imaging was repeated after 6–8 weeks. Before and during therapy, the patients were clinically monitored as recently recommended (Kähler et al. 2016). Follow-up data were collected using chart review and contacting patients, relatives, and resident practitioners and dermatologists if necessary. As controls, we included sex–age-matched healthy subjects as well as non-metastatic CM patients in stage I and II.

### Laboratory parameters

PIV was calculated from absolute values of complete blood counts as follows: (neutrophils (10^3^/mm^3^) × platelets (10^3^/mm^3^) × monocytes (10^3^/mm^3^))/lymphocytes (10^3^/mm^3^). The SII was defined as follows: SII = P × N/L, where P, N, and L were the pre-therapeutic peripheral blood platelet, neutrophil, and lymphocyte counts in cells/L (Yang et al. 2018; Fucà et al. 2020). We evaluated PIV and SII at baseline before the start of ICI therapy and at time of progression/death if applicable.

### Statistics

The MedCalc (Ostende, Belgium) software version 20.009 was used for statistical analysis. Analysis of data distribution was performed by the D’Agostino–Pearson test. For non‐normally distributed variables, the Kruskal–Wallis ANOVA, including Conover post hoc test, and the Wilcoxon test (paired samples) were used. Correlations were assessed using Spearman’s rank correlation procedure. Moreover, receiver operating characteristics (ROC) analyses, including the area under the curve (AUC) and the Youden index, were performed to determine optimal cut-off values. *P* < 0.05 was considered significant.

## Results

**Fig. 1:**
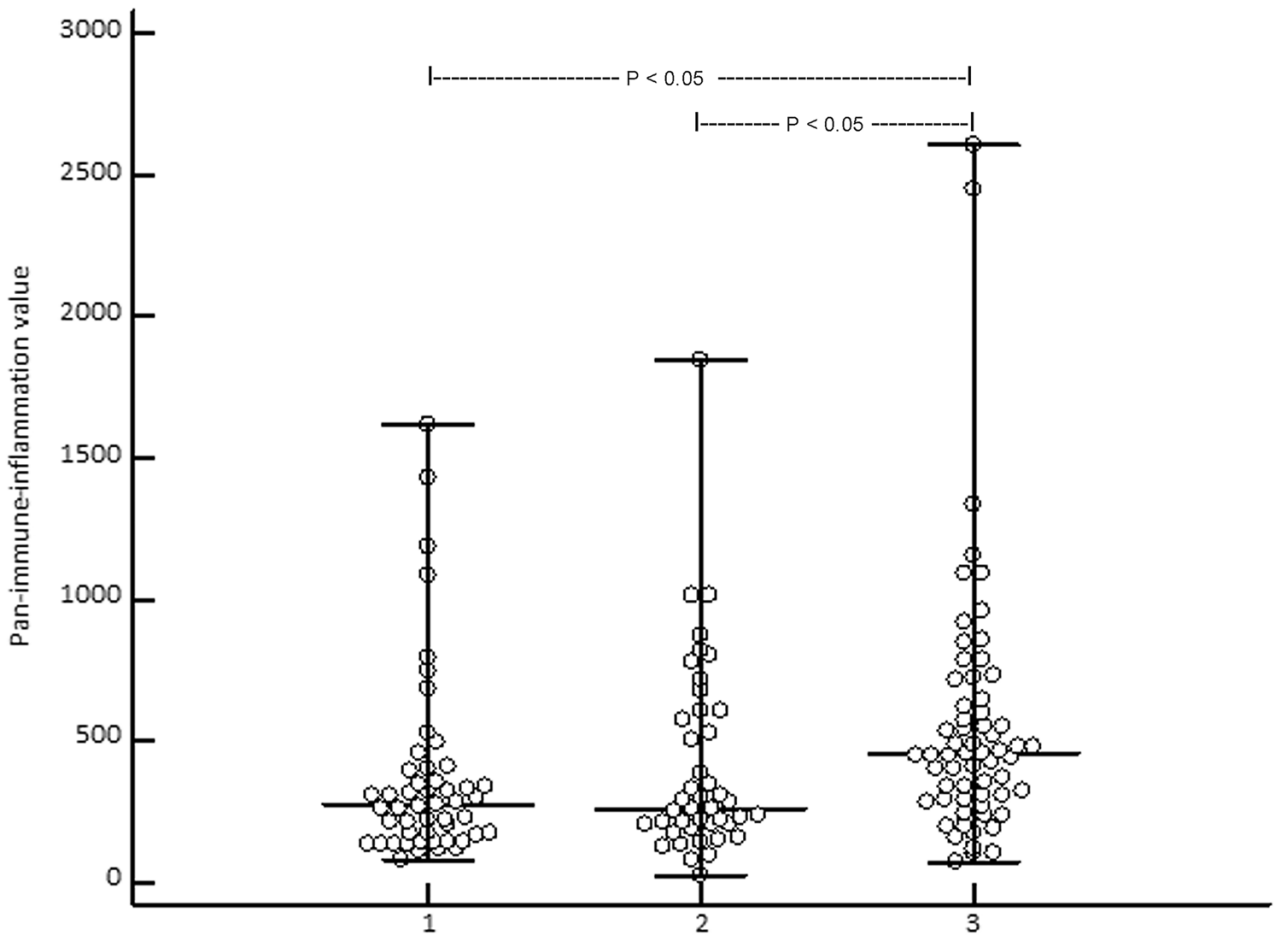
Showing that the median (range) pan-immune-inflammation value is significantly (Kruskal–Wallis ANOVA, P = 0.0011; post-hoc test, P < 0.05) higher in stage III/IV melanoma patients (3) when compared to melanoma patients in stage I/II (2) and healthy controls (1)

Study population consisted of 62 patients with CM, including 22/62 (35.5%) women and 40/62 (64.5%) men at the median age of 67 years (18–85 years). According to the AJCC 8th edition 12/62 (19.4%), patients were in unresectable stage III and 50 (80.6%) were in stage IV prior to the start of ICI treatment (Table 1). In 17/62 (27.4%), a BRAF mutation was found. 24/62 (38.7%) patients received nivolumab, 15/62 (24.5%) pembrolizumab, 9/62 (14.5%) ipilimumab, and 14/62 (22.6%) nivolumab plus ipilimumab. The patients received on median 8 cycles ICI (range: 2–46 cycles). In 17/62 (27.9%) patients, a partial or complete response was observed. Best response according to RECIST 1.1 was observed in 34/62 (54.8%) patients. The median progression-free survival (PFS) was 5 months (range: 3–31 months). A median 5-year melanoma-specific survival (MSS) of 19 months (range: 6–145 months) was observed corresponding to 18/62 (29%) melanoma-specific death events (Fig. 1). Immune-related adverse events of any grade were observed in 20/62 (32.3%) patients. The controls included 43 patients [age: 65 years (17–80); 17 females (39.5%), 26 males (60.5%)] with melanoma in stage I (*n* = 26 (60.5%)) and stage II (*n* = 17 (39.5%)) and 50 healthy subjects [HC, age: 61 years (22–82); 20 females (40%), 30 males (60%)]. With respect to age and gender, there was no significant difference between the three groups as indicated by *P* values > 0.1.

**Table 1:** Overview on baseline characteristics and clinical outcome of patients with advanced melanoma under immune checkpoint inhibitor (ICI) therapy

Parameters	Data of 62 patients in total
Age median (range)	67 (18–85) years
Gender	
Female/male	22 (35.5%)/40(64.5%)
Melanoma stage, S100B, and LDH* prior to ICI	
Inoperable stage III	12 (19.4%)
Stage IV	50 (80.6%)
S100B elevated: no/yes	26 (53.1%)/23 (46.9%)
LDH elevated: no/yes	33 (67.3%)/16 (32.7%)
Median (range) number of ICI cycles	8 (2–46)
irAEs** (no/yes)	42 (67.7%)/20 (32.3%)
Best response	34 (54.8%)
Median (range) progressive-free survival	5 (3–31) months
Melanoma-specific deaths	18 (29%)
Median (range) 5-year melanoma-specific survival	19 (6–145) months

*Lactate dehydrogenase; **Immune-related adverse events

**Fig. 2:**
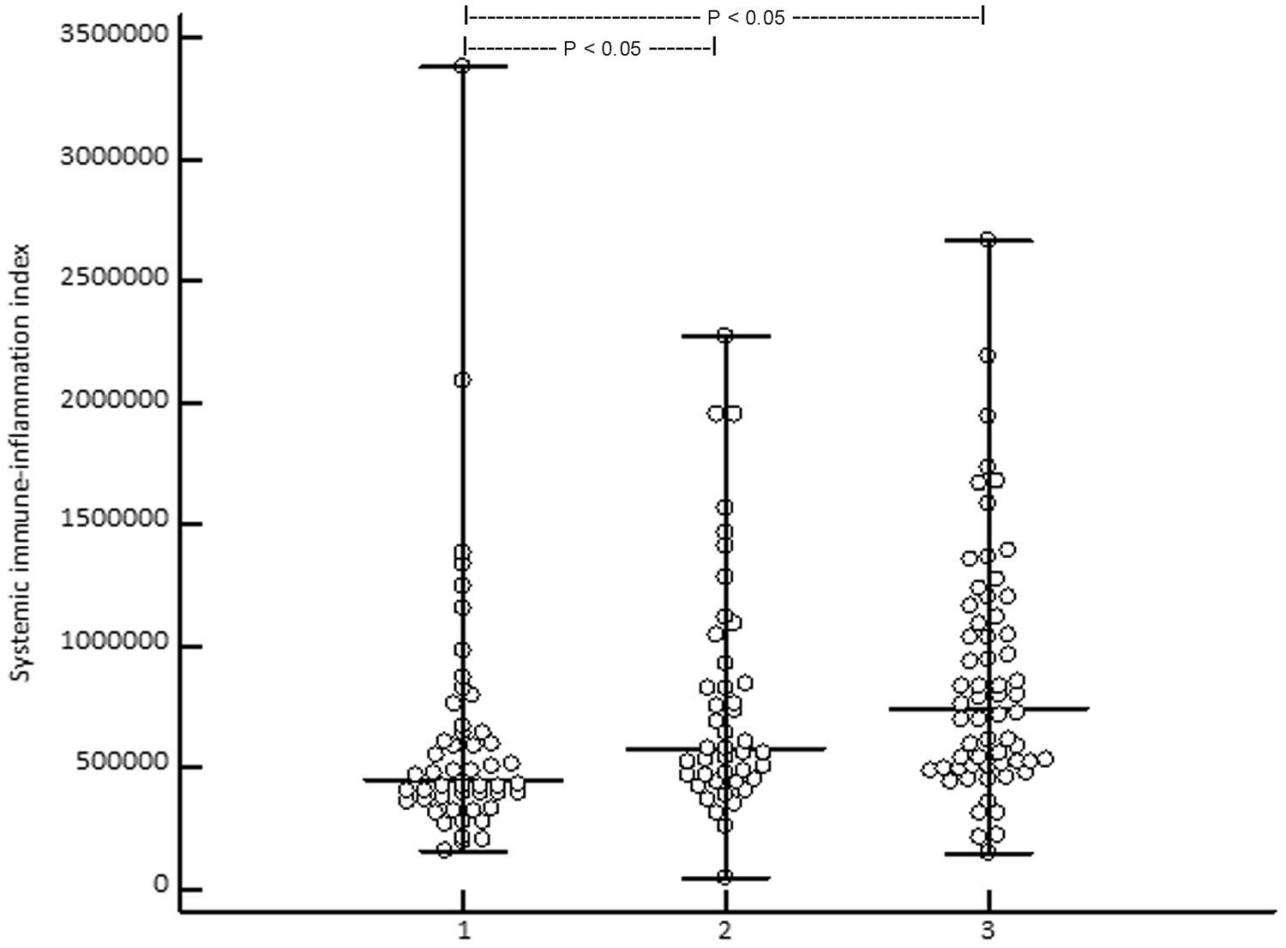
Showing that the median (range) systemic immune-inflammation index is significantly (Kruskal-Wallis ANOVA, P = 0.00044; post-hoc test, P < 0.05) higher in stage III/IV (3) and stage I/II (2) melanoma patients when compared to healthy controls (1)

**Fig. 3:**
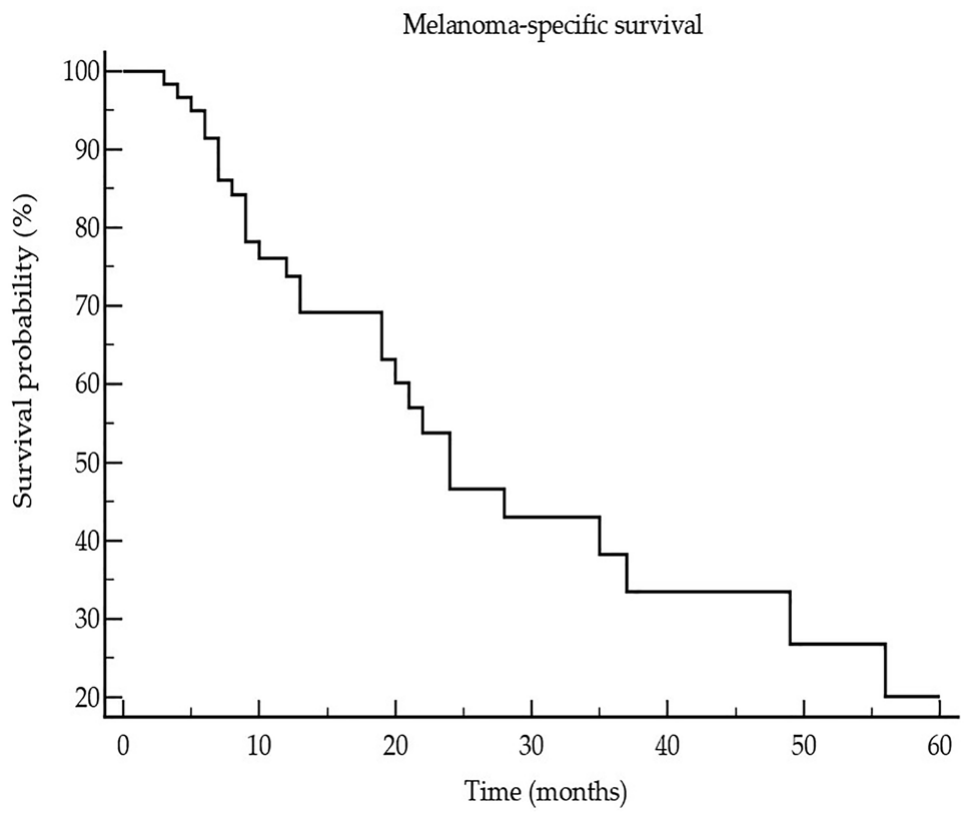
Kaplan–Meier curve showing the 5-year survival of 62 advanced melanoma patients who had undergone treatment with immune checkpoint inhibitors

Kruskal–Wallis ANOVA revealed that the median (range) PIV of stage III/IV patients 455 (74–2611) was significantly (*P* = 0.0011) higher than in CM patients in stage I/II [262 (21–1844)] and HC [272 (81–1622)] (Fig. 2). SII was significantly (*P* = 0.00044) lower in HC [448 × 10^9^/L (159–3.378)] than in stage I/II [580 × 10^9^/L (440–2.276)] and stage III/IV patients [744 × 10^9^/L (147–2.665)] (Fig. 3). In stage III/IV patients, SII positively correlated with age (*r* = 0.33, *P* = 0.0081). At baseline, PIV and SII did not correlate with serum S100B (P = 0.16 and 0.092, respectively). However, lactate dehydrogenase (LDH) significantly correlated with PIV and SII at baseline (*P* = 0.045 and 0.017, respectively). Overall, PIV and SII highly significantly correlated with each other (*r* = 0.90, *P* < 0.0001).

ROC curve statistics revealed that SII (AUC 0.64 (95% CI 0.51–0.76), *P* = 0.13, Youden index 0.38, sensitivity 44%, specificity 93%) as well as PIV (AUC 0.63 (95% CI 0.50–0.75), *P* = 0.17, Youden index 0.32, sensitivity 39%, specificity 93%) were not significantly associated with melanoma death. Moreover, there was no significant association between the biomarkers and other clinical parameters, including progression-free survival (*P* = 0.73 and 0.91, respectively), best response (*P* = 0.87 and 0.64), and immune-related adverse events (*P* = 0.62 and 0.97). There was no significant change of PIV and SII from baseline to progression/death (*P* = 0.38 and *P* = 0.52).

## Discussion

It is well known that systemic inflammation plays a crucial role in tumor development, progression, and metastasis (Liu et al. 2015). Pro-tumorigenic cytokines secreted by neutrophils and platelets, including vascular endothelial growth factor, tumor necrosis factor-α, and interleukin-10, can contribute to cancer progression. Moreover, monocytes as well as lymphocytes have anti-tumoral effects by increasing immune responses against the tumor (Mirili et al. 2019). Recently, systemic immune-inflammation prognosis scores, including NLR, PLR, MLR, and SII, have been reported to be of prognostic value in many malignancy types including melanoma (Templeton et al. 2014; Liu et al. 2015; Nishijima et al. 2015; Zaragoza et al. 2016; Zhong et al. 2017; Kanatsios et al. 2018; Marconcini et al. 2018; Wade et al. 2018; Mirili et al. 2019; Robinson et al. 2020; Bai et al. 2021; Hernando-Calvo et al. 2021; Ludwig et al. 2021). SII represents a promising biomarker in cancers, such as hepatocellular cancer, pancreas, small and non-small cell lung cancer, and gastric and esophageal cancers (Templeton et al. 2014; Yu et al. 2017; Zhong et al. 2017; Yang et al. 2018). Ludwig et al. studied SII in patients with uveal melanoma (*n* = 54) and found that among other factors, low baseline SII was significant independent predictor for prolonged overall survival (Ludwig et al. 2021). Similarly, the predictive power of SII has been reported for patients with high-risk acral melanoma under high-dose interferon therapy, i.e., a low SII (< 615 × 10^9^/l) was associated with a longer relapse-free and overall survival (Yu et al. 2017). In the present report, however, there was no significant correlation between SII and survival parameters. Specifically, while we demonstrated that SII is significantly higher in patients with CM compared to HC, indicating generally enhanced systemic immune-inflammation responses in these tumor patients, we did not observe a significant association between SII and clinical outcome parameters, such as response to ICI treatment and PFS and MSS. In line with this finding, Mirili et al. did not observe on multivariate analyses that SII was an independent predictor for overall survival in patients with CM (*n* = 101) (Mirili et al. 2019).

Fest et al. recently studied prognostic inflammatory markers such as SII and found that these markers increase with age. Indeed, in our cohort of stage III/IV patients SII positively correlated with age (*r* = 0.33, *P* = 0.0081), which was not the case for stage I/II CM patients as well as HC (Fest et al. 2018). In CM patients with metastatic disease, there may exist additional immune-inflammation responses which increase with age. Li et al. suggested that SII is a robust indicator of tumor differentiation and one-year survival in elderly patients with newly diagnosed solid tumors (Li et al. 2018). They found that patients in the high SII group showed poor tumor differentiation and poor prognosis compared to patients with a low SII score (Li et al. 2018). Similarly, sex-dependent differences of SII have been reported in patients suffering from different cancers (Fest et al. 2018; Li et al. 2018). Consequently to avoid these confounders, we matched the groups investigated with respect to age and sex.

Fucà et al. recently reported for the first time on the prognostic role of the novel immune-inflammatory, blood-based biomarker score — the PIV — that integrates neutrophil, platelet, monocyte, and lymphocyte counts in patients with metastatic colorectal and breast cancer. In a retrospective analysis of metastatic melanoma patients treated with first-line ICI (*n* = 119) or targeted therapy (*n* = 109), Fucà et al. could show that a high baseline PIV (> 600) was independently associated with poor PFS and overall survival. Moreover, Fucà et al. also observed that a high PIV was associated with primary resistance to both ICI and targeted therapy. However, Fucà et al. did not study PIV in other melanoma stages or HC (Fucà et al. 2020). In line with their finding that an elevated PIV correlated with higher M stage and elevated LDH, we observed that the median PIV of stage III/IV CM patients is significantly higher than that of patients with stage I/II or HC. Thus, PIV may also represent a surrogate marker for tumor burden. Thus, it was surprising that we could not detect a significant correlation between PIV and clinical outcome measures, such as treatment response, PFS and MSS as reported Fucà and coworkers (Fucà et al. 2021). This discordance between the studies may be explained by differences in sample sizes or the fact that we included a relatively large proportion of patients with unresectable stage III melanomas, whereas the study of Fucà et al. exclusively included stage IV CM patients (Fucà et al. 2021).

In conclusion, we demonstrated for the first time that the systemic immune-inflammation biomarker PIV is significantly higher in patients with metastatic CM when compared to HC and stage I/II melanoma patients. By contrast, SII appears to be better suitable to differentiate between CM patients and HC. Even though both immune-inflammation biomarkers showed some power to differentiate between CM stages and HC, respectively, PIV and SII seem not to be significant predictors for clinical outcome measures of CM patients under ICI therapy. However, the true predictive power of PIV and SII has to be studied in larger prospective investigations on patients with CM.

## Data Availability

Derived data supporting the findings of this study are available from the corresponding author on reasonable request.
